# Unexpected Vertical Transmission of SARS-CoV-2: Discordant Clinical Course and Transmission from Mother to Newborn

**DOI:** 10.3390/microorganisms10091718

**Published:** 2022-08-26

**Authors:** Alessandra Boncompagni, Mattia De Agostini, Licia Lugli, Giliana Ternelli, Valeria Colonna, Emanuela Biagioni, Maria Paola Bonasoni, Tiziana Salviato, Liliana Gabrielli, Mirella Falconi, Fabio Facchinetti, Alberto Berardi

**Affiliations:** 1 Neonatal Intensive Care Unit, Department of Medical and Surgical Sciences of Mothers, Children and Adults, University Hospital of Modena, 41124 Modena, Italy; 2 Pediatric Unit, Department of Medical and Surgical Sciences of Mothers, Children and Adults, Post-Graduate School of Pediatrics, University of Modena and Reggio Emilia, 41124 Modena, Italy; 3 Obstetrics and Gynecology Unit, Department of Medical and Surgical Sciences of Mothers, Children and Adults, University Hospital of Modena, 41124 Modena, Italy; 4 Intensive Care Unit, University Hospital of Modena, 41125 Modena, Italy; 5 Pathology Unit, Azienda USL-IRCCS di Reggio Emilia, 42122 Reggio Emilia, Italy; 6 Pathology Institute, University of Modena and Reggio Emilia, 41121 Modena, Italy; 7 Microbiology Unit, IRCCS Azienda Ospedaliero-Universitaria di Bologna, 40126 Bologna, Italy; 8 Department of Experimental, Diagnostic and Specialty Medicine, University of Bologna, 40138 Bologna, Italy

**Keywords:** SARS-CoV-2, COVID-19, vertical transmission, newborn, placenta, infection, histopathological findings

## Abstract

Mother-to-newborn COVID-19 transmission is mainly postnatal, but single-case reports and small case series have also described SARS-CoV-2 transplacental transmission. Unfortunately, studies regarding vertical transmission of SARS-CoV-2 lack systematic approaches to diagnosis and classification. So far, scientific evidence seems to suggest that the severity of maternal infection increases the risk of vertical transmission. We report two neonates born from COVID-19-positive mothers, of which one of the newborns had a vertical infection. The placental involvement, and consequent intrauterine transmission of SARS-CoV-2, were inversely related to the severity of the maternal disease. The description of cases divergent from current evidence on this topic could provide new insights to better understand SARS-CoV-2 vertical transmission.

## 1. Introduction

In March 2020, the World Health Organization (WHO) declared COVID-19 disease a pandemic caused by the novel severe acute respiratory syndrome coronavirus 2 (SARS-CoV-2). The epidemiology of this disease is changing over time, but it is known that COVID-19 can affect individuals from birth to the elderly, with different degrees of severity and variable involvement of organs and systems [1,2,3].

Information about its impact on pregnancy and neonates has partially changed from the beginning of the pandemic [4,5,6,7,8,9]. Around the world, recommendations on breastfeeding and maternal–infant contact may vary from different scientific societies, but often maternal breastfeeding and nonseparation of mothers and neonates are suggested. Perinatal infection is rare and frequently paucisymptomatic [10,11,12,13]. Mother-to-child transmission of SARS-CoV-2 can potentially occur in different moments according to the WHO’s categorization [14]: intrauterine (through the hematogenous route, or more rarely, the ascending route); intrapartum (during labor and childbirth); or early postnatal (through breastfeeding, contact, respiratory or other infectious maternal secretions). Even if the predominant mode of mother-to newborn transmission is postnatal, single-case reports or small case series have described SARS-CoV-2 transplacental transmission [7,15,16,17,18]. However, many aspects of its clinical impact are still unclear.

To the best of our knowledge, all previously reported cases of confirmed intrauterine SARS-CoV-2 transmission were born to symptomatic mothers. We report two neonates born from COVID-19-positive mothers, of which one of the newborns had a vertical infection. The placental involvement, and consequent intrauterine transmission of SARS-CoV-2, were inversely related to the severity of the maternal disease. These findings could provide new insights on the transplacental transmission of SARS-CoV-2.

## 2. Case Description

### 2.1. Case 1

A North African 34-year-old woman, gravida1, para 0, was admitted to the hospital at 28 weeks’ gestation with fever, dyspnea and cough. SARS-CoV-2 infection was detected 8 days before admission in an otherwise uncomplicated pregnancy. The maternal medical history was silent, except for nephrotic syndrome in remission. At admission, she underwent blood exams (main results in Table 1) and was given antibiotics along with anticoagulant therapy. The chest X-ray was consistent with bilateral interstitial pneumonia, and respiratory support was started for low oxygen saturation. However, respiratory failure worsened, and she was eventually admitted to the intensive care unit, where antiviral and monoclonal antibody therapy were given. On the fourth day after admission, emergency cesarean section was performed because of abnormal cardiotocographic (CTG) trace. 

A male newborn was delivered (gestational age 28^+3^ weeks; birth weight 1317 g, 86th percentile; length 35 cm, 19th percentile; cranial circumference 26 cm, 53rd percentile). The Apgar score was 3–5–5 at 1–5–10 min, respectively. The newborn was intubated at birth and admitted to the neonatal intensive care unit (NICU) in the isolation room.

**Figure 1:**
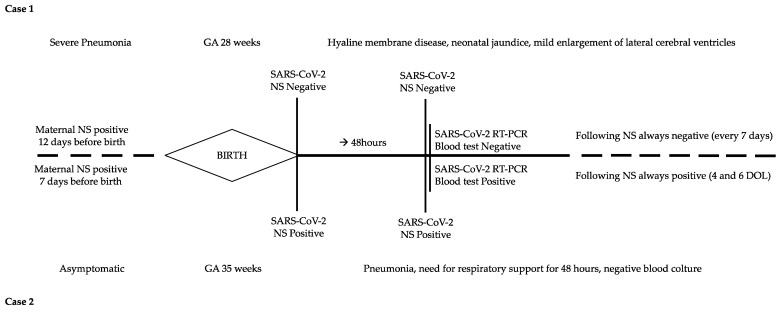
Timeline of clinical course and laboratory tests of the two cases. DOL: day of life; GA: gestational age; NS: nasopharyngeal swab.

At admission, cord blood gas analysis revealed a mild metabolic acidosis (pH 7.26, BE −6.6 mmol/L, pCO2 45 mmHg) that resolved spontaneously. The first chest X-ray was consistent with hyaline membrane disease; therefore endotracheal surfactant was administered. A few hours later, the newborn was extubated and underwent noninvasive ventilation. Empirical broad-spectrum antibiotics were discontinued (at 48 h of life) when results of blood culture were available. Blood tests were within a normal range and all real-time polymerase chain reaction (RT-PCR) assays for SARS-CoV-2 infection were negative (Table 1, Figure 1); on day of life (DOL) 2, the newborn was treated with phototherapy for moderate jaundice. Because of the severe maternal clinical conditions, the newborn was formula-fed. The noninvasive respiratory support was successfully discontinued 24 days after birth; clinical course was regular for a premature newborn, and the infant was healthy-appearing when discharged home (35 weeks post-menstrual age—PMA). Brain MRI at term-corrected age showed mild enlargement of lateral ventricles. Neurodevelopmental follow-up at 6 and 12 months corrected age was within normal range and no hearing impairment was diagnosed at the follow-up audiologic tests. 

### 2.2. Case 2

An Italian 38-year-old woman, gravida 2, para 1, was admitted to the hospital at 35 weeks’ gestation because of reduced fetal movements. The nasopharyngeal swab for SARS-CoV-2 was positive 7 days before hospital admission, but she was asymptomatic. Her medical history was uneventful; investigations carried out during pregnancy were all within a normal range. On admission, blood exams showed an increased D-dimer (>40,000 ng/mL) and emergency cesarean section was performed due to signs of fetal distress.

A female newborn was delivered (gestational age 35^+5^ weeks; birth weight 2089 g, 25° percentile; length 45 cm, 37th percentile; head circumference 31 cm, 30th percentile). The Apgar score was 6–7–9 at 1–5–10 min, respectively. At birth, the newborn required nCPAP (FiO_2_ 0.25) for respiratory failure and was then admitted to NICU in the isolation room. Blood gas analysis at 1 h of life showed a mild increase in negative base excess (pH 7.28, BE −11.5 mmol/L, pCO_2_ 29.6 mmHg), which improved spontaneously in the next hours. Wide-spectrum empirical antibiotics were administered for 48 h. Repeated RT-PCR on nasopharyngeal swabs and blood tests were positive for SARS-CoV-2 (Table 1, Figure 1). nCPAP was discontinued 48 h after birth and breast milk was administered.

The clinical condition of the baby transiently worsened at DOL 5: the blood culture was sterile and the C-reactive protein was negative, while the chest X-ray showed a small right paracardiac opacity that improved within few days. The newborn was eventually discharged home healthy-appearing at DOL 10 (37 weeks PMA).

## 3. Placental Pathology

**Figure 2:**
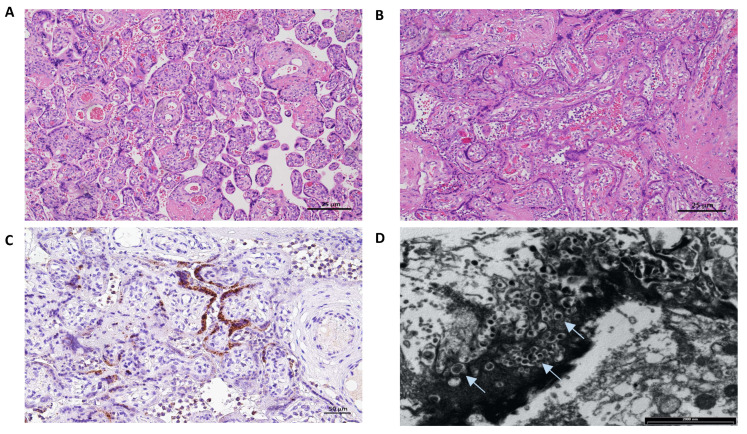
Placental pathology findings of the two cases. **The Placenta of case 1** weighed 246 g and measured 13 × 12 × 4 cm; the umbilical cord appeared hypercoiled (5 coils per 10 cm) and was 13 cm long with a diameter between 0.9–1.5 cm. No gross anomalies were observed. (**A**) Hematoxilin and Eosin staining (4HPF): mild subchorionitis with normally developed parenchyma for gestational age. **The Placenta of case 2** weighed 347 g and measured 18 × 13 × 3 cm; the umbilical cord had 2 coils every 10 cm and was 27 cm long with a diameter between 1–1.5 cm. Macroscopically, it appeared pale. (**B**) Hematoxilin and Eosin staining (4HPF): diffuse intervillous fibrinoid deposition with intervillositis mainly represented by lymphocytes, histiocytes and few granulocytes. (**C**) Immunostaining for SARS-CoV-2 nucleocapsid protein (polyclonal, Novus biological, USA) (10HPF): diffuse positivity in the syncytiotrophoblast. Lymphocyte immunohistochemical characterization (not shown) revealed a prevalence population of TCD4, and few activated TCD8 evidenced by Granzyme B staining. (**D**) Electron microscopy examination: many viral particles (arrows) in the cytoplasm of the syncytiotrophoblast.

Placental analyses included optic and electron microscopy (EM), immunohistochemistry and SARS-CoV-2 detection with qualitative and quantitative RT-PCR on paraffin-embedded tissues. The main results are summarized in Table 1 and Figure 2. The placenta of case 2 was macroscopically pale, and showed histologically intervillous fibrinoid deposition with intervillositis, while the immunostaining for SARS-CoV-2 revealed diffuse positivity in the syncytiotrophoblast and a prevalence population of TCD4 lymphocytes. Electron microscopy revealed many viral particles (arrows) in the cytoplasm of the syncytiotrophoblast.

## 4. Discussion

Similar to previous coronavirus infections, intrauterine transmission of SARS-CoV-2 was considered unlikely at the beginning of the pandemic [4,19], but evidence of potential perinatal transmission was later demonstrated [15,18]. Transplacental infection from mother to fetus may occur with different possible mechanisms: direct villous tree damage, transmission from maternal endothelium to the extravillous trophoblast, involvement of maternal immune system with transcellular transport, and more rarely, infection through the vagina [20]. However, in most cases, the transmission remains unproven because of a lack of information about RT-PCR tests on newborn samples or the placenta.

Proven vertical transmission of SARS-CoV-2 has been described in a few cases (0.5–5%, depending on different studies), mostly in the third trimester of pregnancy [16]. Nowadays, evidence of vertical transmission and placental involvement is growing [17,21].

Some studies and review reports have investigated the effects of COVID-19 on the placenta, trying to correlate maternal status, placental involvement and vertical transmission to neonates [16,20,22,23]. Even so, the scientific literature necessitates being more homogeneous and comparable in terms of classification of mother-to-newborn transmission, definitions, timing and specimens tested.

We report two cases occurring at our hospital in the same month (April 2021), to emphasize their opposite clinical courses of maternal SARS-CoV-2 infection during late pregnancy and the unexpected transmission to one of the two newborns. The case 1 mother was severely symptomatic with high SARS-CoV-2 viremia. In this case, transplacental transmission did not occur, and neonatal SARS-CoV-2 tests were persistently negative, although we do not know whether the severe maternal inflammatory state damaged the vulnerable cerebral white matter of the preterm infant, as occurs in maternal chorioamnionitis [24]. In contrast, the case 2 mother was fully asymptomatic at delivery, but the placenta was highly positive for SARS-CoV-2. According to the WHO classification, this second case was a confirmed intrauterine transmission. Repeated neonatal nasopharyngeal swabs and positive RT-PCR in blood confirmed the transmission of SARS-CoV-2 to the neonate, who had a mild disease, as often happens in young infants [18].

Only in a few previously reported cases [15,25] has transplacental transmission been confirmed; because of a lack of specific analyses, transplacental transmission remains only probable in most reported cases [18,25]. The placentas of pregnant women (with or without clinical symptoms) with COVID-19 infection depict a wide spectrum of pathological findings [22,23]. These findings include signs of inflammation with villous infiltration and intervillitis, vascular malperfusion and involvement of syncytiotrophoblast cells or other fetal cells [20]. Nonetheless, no specific or pathognomonic placental lesions have been identified for SARS-CoV-2 infection [26,27]. The transplacental transmission to the newborn is rare even when the placenta is positive for SARS-CoV-2, and investigators have postulated different models to explain this protective effect [20,28]. When intrauterine transmission to the newborn is confirmed, the placentas from infected maternal–neonatal dyads are characterized by chronic histiocytic intervillositis, together with massive perivillous fibrin deposition and syncytiotrophoblast necrosis [20,22,23]. This lesion was generally identified as “SARS-CoV-2 placentitis” [29]. Despite its potential association with miscarriage or stillbirth, vertical transmission to the newborn has not always been clarified, even in case of tissue positivity for COVID-19 [29,30,31]. The pathophysiological mechanism underlying this lesion is still unclear. Chronic histiocytic intervillositis, intervillous fibrin deposition, and trophoblast necrosis cascade may be activated by a compound effect of a maternal and/or fetal dysregulation of the immune response and an intrinsic procoagulant status [31]. Before COVID-19, it was well-known that chronic histiocytic intervillositis had an immunological basis and a high risk of recurrence, even though its combination with intervillous fibrin deposition was occasionally reported [32,33]. In addition, chronic histiocytic intervillositis and intervillous fibrin deposition have been related to intrauterine fetal death, early-onset intrauterine growth restriction and newborn neurological symptoms [33]. However, in two wide recent studies, “SARS-CoV-2 placentitis” has been found to have a very low incidence and no significant association with neonatal morbidity and/or intrauterine growth restriction [26,31]. So far, how this lesion may affect the fetus has yet to be elucidated, and the timing of the maternal viral infection may play a key role in determining newborns’ outcomes.

Regarding the cases we described, to the best of our knowledge, all previously reported cases of intrauterine transmission were associated with relevant maternal symptoms [25]. Vertical transmission was attributed to the prominent maternal inflammatory status, leading to placental damage and consequent transplacental transmission through syncytiotrophoblast. In case 2, maternal blood tests showed high levels of D-dimer, but it is still unknown if this finding plays a role in promoting vertical transmission. As mentioned before, an abnormal maternal procoagulant condition associated with a dysregulation of the immune system may explain the raised levels of D-dimer and intervillous fibrin deposition with chronic intervillositis [31]. Furthermore, this condition has been mainly connected with severe or critical COVID-19 disease and a consequent cytokine storm with pathological implications both for the mother and the baby.

Our case underlines that vertical transmission is not yet fully understood. Maternal status does not necessarily correlate neither with placental pathological findings nor with the probability of intrauterine transmission to the newborn. However, consistent with the previous literature, neonates with a positive SARS-CoV-2 test usually remain only mildly symptomatic, even if the placenta is highly damaged; a protective role of breastfeeding could be hypothesized in some cases [34].

In conclusion, this is the first case reporting the transplacental transmission of SARS-CoV-2 from an asymptomatic mother who had relevant placental pathological findings. The vertical transmission of SARS-CoV-2 requires further investigation with larger studies and a standardized approach for comparing results and drawing possible conclusions on neonatal outcomes.

## Figures and Tables

**Table 1:** Maternal and neonatal data of the two cases.

Data	Case 1	Case 2
**Maternal characteristics**		
Ethnic Group	North African	Caucasian
Pre-existing medical conditions	Nephrotic syndrome in remission	None
BMI	<25	<25
Sociodemographic risk factors	NA	NA
Smoking	No	No
Pregnancy course	Regular	Regular
Antenatal exams	Normal	Normal
Maternal symptoms	Severe pneumonia	Asymptomatic
**Maternal laboratory findings**		
Platelets (109/L)	303	130
Hemoglobin (g/dL)	9.8	12.2
LDH (U/L)	1045	1313
D-dimer (ng/dL)	1440	>40,000
CRP (mg/dL)	13.3	3.4
IL-6 (pg/mL)	326.1	NA
Blood SARS-CoV-2 (copies/mL)	1066	NA
**Neonatal characteristics**		
Signs/symptoms	Hyaline membrane disease	Pneumonia
**Neonatal SARS-CoV-2 investigations** **(Real-time polymerase chain reaction, RT-PCR)**		
Nasopharyngeal swab at birth	Negative	Positive
Nasopharyngeal swab at 48 h of life	Negative	Positive
Nasopharyngeal swab until discharge	Every 7 days, always negative	Positive at DOL 4 and 6
Blood (copies/mL)	Negative	1108
**Placental examinations**		
RT-PCR SARS-CoV-2 (copies/µg)	Negative	1,068,963

BMI: body mass index (kg/m^2^); CRP: C-reactive protein; DOL: day of life; IL-6: interleukin-6; LDH: lactate dehydrogenase; NA: not assessed; RT-PCR: real-time polymerase chain reaction.
